# A Case Report of Robotic Salvage Tumor Resection with Combined Resection of the Internal Iliac Vessels and Ureter for Lateral Local Recurrence after Lateral Lymph Node Dissection for Rectal Cancer

**DOI:** 10.70352/scrj.cr.26-0126

**Published:** 2026-06-03

**Authors:** Riki Ohno, Haruka Oi, Soichiro Natsume, Kazuki Kawasaki, Yuichiro Yoshioka, Toshiya Nagasaki

**Affiliations:** Department of Gastroenterological Surgery, Saitama Cancer Center, Kitaadachi, Saitama, Japan

**Keywords:** lateral local recurrence, locally recurrent rectal cancer, robotic surgery

## Abstract

**INTRODUCTION:**

Lateral local recurrence (LLR) is a recognized pattern of local recurrence in patients with low rectal cancer (LRC) following curative resection, and salvage surgery for LLR is technically challenging. Recent studies have reported the feasibility of robotic lateral lymph node dissection (LLND) in patients with LRC; however, data regarding the safety and usefulness of robotic salvage surgery for LLR after LLND remain limited. Herein, we report a case of robotic salvage tumor resection with combined resection of the internal iliac vessels (IIVs) and ureter for LLR following LLND.

**CASE PRESENTATION:**

A 65-year-old man underwent low anterior resection with LLND for LRC without preoperative treatment. The pathological diagnosis was pT3N1bM0, stage IIIB, and adjuvant chemotherapy was administered. LLR was detected 4 years after the initial surgery. Following preoperative long-course chemoradiotherapy (pre-CRT), the patient underwent robotic tumor resection with combined resection of the IIVs and ureter. Ureteroneocystostomy with psoas hitch was subsequently performed. The lumbosacral nerve trunk and sacral plexus were preserved. The operative time was 295 min, and the estimated blood loss was 40 mL. A negative resection margin was obtained. The patient remained recurrence-free at 6 months of follow-up.

**CONCLUSIONS:**

This case demonstrates that robotic salvage surgery for LLR following LLND is feasible and can achieve oncologically adequate resection in a minimally invasive modality. The combination of the technical advantages of robotic surgery and tumor regression following pre-CRT may facilitate an optimal balance between oncological radicality and functional preservation.

## Abbreviations

IIVs
: internal iliac vessels
LLND
: lateral lymph node dissection
LLR
: lateral local recurrence
LRC
: low rectal cancer
pre-CRT
: preoperative long-course chemoradiotherapy

## INTRODUCTION

The Japanese guideline recommends bilateral LLND for patients with locally advanced LRC. Following LLND, lateral pelvic structures, including the obturator nerve, internal obturator muscle, and IIVs, become exposed. LLR has been reported to occur in approximately 5.5% of cases after LLND.^1)^ Salvage surgery for LLR following LLND is technically challenging because combined resection of these exposed structures is often required. Recent studies have reported the feasibility of robotic LLND in patients with LRC^2)^; however, there are almost no reports evaluating the safety and usefulness of robotic salvage surgery for LLR after LLND.

Herein, we report a case of robotic salvage tumor resection with combined resection of the IIVs and ureter for LLR following LLND. This report is presented in accordance with the Surgical CAse REport (SCARE) criteria.^3)^

## CASE PRESENTATION

A 65-year-old man with a history of diabetes mellitus and hypertension presented with locally advanced LRC. He underwent laparoscopic low anterior resection with LLND and ileostomy. The pathological diagnosis was pT3N1bM0, stage IIIB. A total of 7 lateral lymph nodes were dissected on the right side and 11 on the left, with no evidence of metastasis. Adjuvant chemotherapy consisting of 8 cycles of capecitabine plus oxaliplatin was administered. Stoma closure was performed 8 months after the initial surgery.

**Fig. 1:**
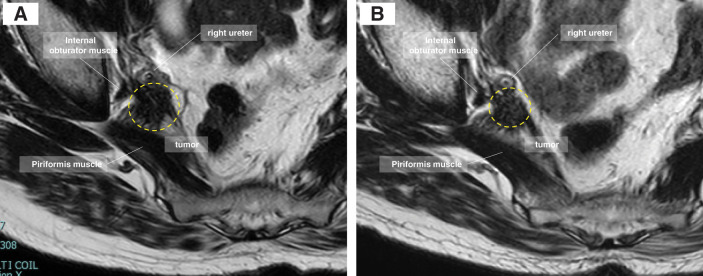
T2-weighted MRI reveals a tumor (dotted circle) with irregular borders and invasion of the right ureter, internal obturator muscle, internal iliac artery, and vein in the right lateral pelvic area. (**A**) Before pre-CRT. (**B**) After pre-CRT. pre-CRT, preoperative long-course chemoradiotherapy

Four years after the initial surgery, serum carcinoembryonic antigen and carbohydrate antigen 19-9 levels increased to 5.5 and 155 ng/mL, respectively. CT revealed no evidence of distant metastasis; however, a tumor was identified in the right lateral pelvic region, accompanied by right-sided hydronephrosis. MRI revealed a tumor with irregular borders and infiltration into adjacent structures, including the right ureter, internal obturator muscle, and IIVs (**Fig. 1A**). Following placement of a right ureteral stent, pre-CRT was performed, followed by radical resection. The tumor size decreased from 25 to 16 mm after pre-CRT (**Fig. 1B**). Six weeks after completion of pre-CRT, robotic salvage surgery was performed.

**Fig. 2:**
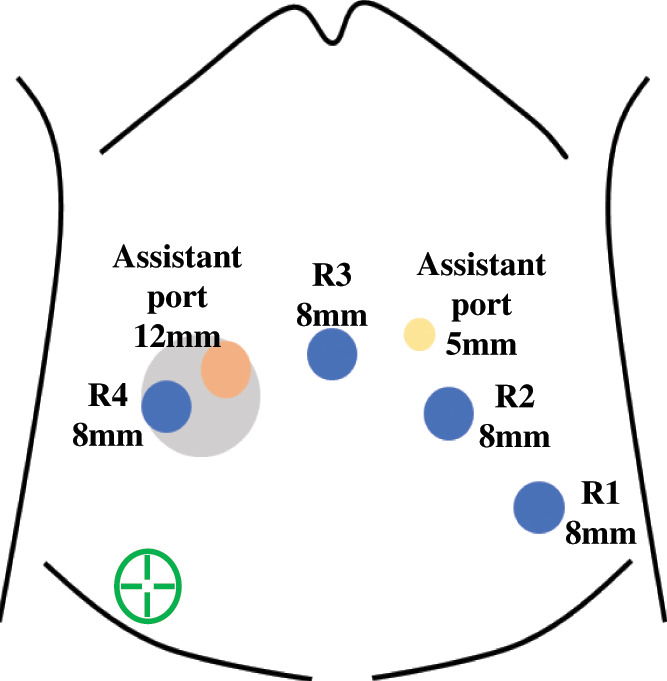
Port placement consisted of a total of 6 ports: 4 da Vinci ports and 2 assistant ports. R, robotic port

**Fig. 3:**
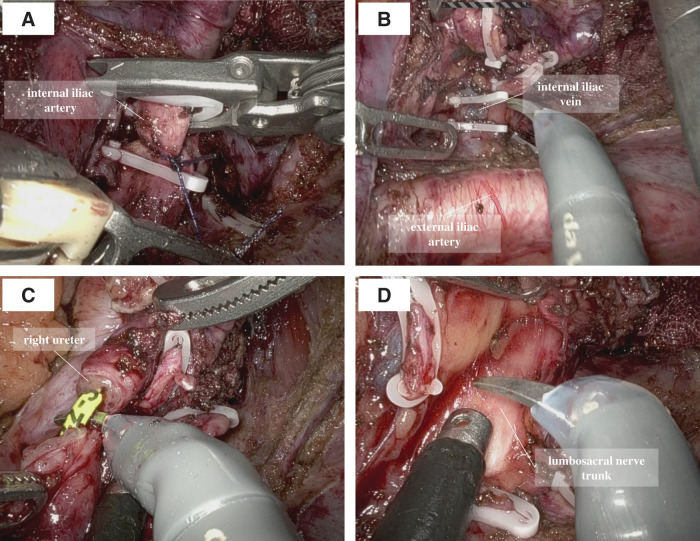
(**A**) Proximal division of the internal iliac artery. (**B**) Proximal division of the internal iliac vein. (**C**) Ureteral division proximal to the tumor invasion. (**D**) Posterior dissection of the tumor along the surface of the lumbosacral nerve trunk.

**Fig. 4:**
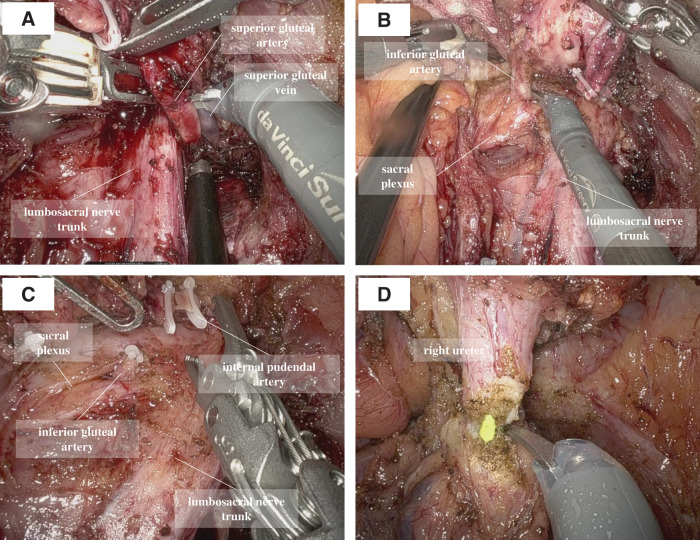
(**A**) Distal division of the superior gluteal artery. (**B**) Distal division of the inferior gluteal artery. (**C**) Distal division of the internal pudendal artery. (**D**) Ureteral division distal to the tumor invasion.

**Fig. 5:**
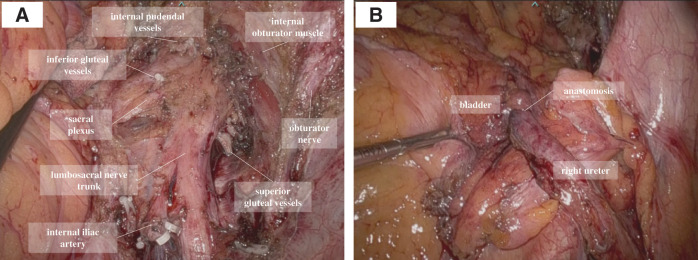
(**A**) Intraoperative image after tumor resection. The lumbosacral nerve trunk and sacral plexus are preserved. (**B**) Urinary tract reconstruction was performed using robotic ureteroneocystostomy with a psoas hitch.

The patient was placed in the Trendelenburg position, and a single-docking technique was used with the da Vinci X platform (Intuitive Surgical, Sunnyvale, CA, USA) positioned between the patient’s legs. Port placement consisted of 6 ports: 4 da Vinci ports and 2 assistant ports (**Fig. 2**). Intraoperatively, the tumor was located in the right lateral pelvic region, and dilation of the proximal ureter was observed. Initial exposure of the external iliac vessels, iliopsoas muscle, internal obturator muscle, and obturator nerve was attempted but was limited due to severe fibrosis. The seminal duct and umbilical artery were divided, and the Retzius space was opened to detect the internal obturator muscle and obturator nerve. After identifying the internal obturator muscle, the muscle adjacent to the tumor was partially resected while preserving the obturator nerve. The proximal sides of the IIVs, ureter, and obturator nerve were successfully exposed. The right ureter and IIVs were divided proximal to the tumor (**Fig. 3A**–**3C**). The surfaces of the lumbosacral nerve trunk and sacral plexus were exposed, and the posterior side of the tumor was dissected along these neural structures (**Fig. 3D**). The superior gluteal vessels were divided at the entrance to the supra-piriformis foramen (**Fig. 4A**), followed by division of the inferior gluteal vessels at the entrance to the infra-piriformis foramen. The internal pudendal vessels were divided distal to the tumor, reaching the surface of the coccygeal muscle (**Fig. 4B** and **4C**). The ureter was divided distal to the tumor, and the vesical vessels were divided at their entrance to the bladder (**Fig. 4D**). The specimen was removed, and urinary tract reconstruction was performed robotically by a urologist using ureteroneocystostomy with a psoas hitch. The ureteral stent was replaced during the procedure (**Fig. 5A** and **5B**). The operative time was 295 min, and the estimated blood loss was 40 mL. The postoperative course was uneventful and the patient was discharged on POD 11. The pathological examination revealed no identifiable lymph node structures in the resected specimen; however, perineural invasion was observed, suggesting tumor spread along neural pathways. A tumor-free resection margin was confirmed. The ureteral stent was removed at 8 weeks postoperatively. At 6 months of follow-up, the patient remained in good health and recurrence-free, with no complications, including urinary tract infection or ureteral stricture.

## DISCUSSION

Salvage surgery for patients with LLR following LLND is technically demanding because of the complex pelvic anatomy, severe fibrotic changes, and loss of adipose tissue surrounding the lateral pelvic structures. To the best of our knowledge, literature reporting robotic salvage surgery specifically for LLR following LLND is extremely limited. In the present case, a relatively short operation time, minimal blood loss, an uneventful postoperative course, and a negative resection margin were observed. These findings suggest that a robotic approach may provide a feasible surgical and oncological option for salvage surgery in patients with LLR. However, this report has a significant limitation, namely, the short postoperative follow-up period of only 6 months. Therefore, further observation is required to evaluate the long-term outcomes.

In the management of local recurrence, achieving R0 resection is the most critical prognostic factor for survival.^4)^ Therefore, careful preoperative assessment of resectability is essential in determining surgical candidacy. In patients who have undergone LLND, anatomical landmarks around the recurrent tumor are often obscured, making identification of appropriate dissection planes particularly challenging. In addition, combined resection of adjacent organs is frequently required to ensure oncological radicality; however, previous studies have reported relatively low R0 resection rates.^5,6)^ Even when R0 resection is technically achievable, there is a risk of irreversible functional impairments, such as gait disturbance resulting from nerve damage, which may significantly compromise postoperative QOL. Therefore, a careful balance between oncological benefit and functional outcome is required, and surgical indications should be determined cautiously with thorough informed consent. In the present case, the patient had an isolated LLR without distant metastasis. Preoperative MRI detected tumor invasion into the ureter, internal obturator muscle, and IIVs, while the lumbosacral nerve trunk and sacral plexus were not invaded. Although the tumor appeared to be in contact with these neural structures, the contact area was very limited, and the absence of secondary signs, such as nerve thickening or signal changes, suggested no direct invasion. Furthermore, tumor shrinkage following pre-CRT was observed. Based on these findings, complete tumor resection was considered achievable while preserving the neural structures. Pre-CRT has been reported to improve R0 resection rates and prognosis in patients with LR.^7)^ In this case, tumor regression following pre-CRT likely contributed to both oncological radicality and functional preservation.

The most important surgical aspect of this case was to ensure the precise dissection between the dorsal side of the tumor and the surface of the lumbosacral nerve trunk. In an open approach, performing sharp dissection in the narrow, deep pelvic space is challenging. Previous studies demonstrated that laparoscopic LLND is feasible and less invasive than laparotomy.^8–10)^ Moreover, a recent report suggested that the robotic salvage surgery for LLR may be associated with reduced intraoperative blood loss than the laparoscopic approach, and conversion to laparotomy was required only in laparoscopic cases with infiltration to the iliac vessels and uncontrollable bleeding from the iliac vessels.^11)^ Certain characteristics of robotic surgery, including a stable 3D view, multi-articulated instruments, and tremor filtration, offer significant advantages during dissection of the lateral pelvic vessels. Therefore, a robotic approach may be particularly beneficial in cases requiring dissection of the IIVs, as in the present case. Nevertheless, salvage surgery for LRC patients with LLR remains technically challenging and carries the risk of serious intraoperative and/or postoperative complications. Accordingly, careful patient selection based on imaging findings, as well as surgical expertise in robotic LLND, is essential.

## CONCLUSIONS

Robotic salvage surgery may represent a promising minimally invasive modality for LLR, even following previous LLND. The technical advantages of robotic surgery, combined with tumor regression following pre-CRT, may facilitate an optimal balance between oncological radicality and functional preservation.

## DECLARATIONS

### Availability of data and materials

The data are not publicly available due to privacy or ethical restrictions. Data are available from the corresponding author upon reasonable request.
